# Di-*tert*-butyl­chlorido(*N*,*N*-dibenzyl­dithio­carbamato)tin(IV)

**DOI:** 10.1107/S1600536811006866

**Published:** 2011-02-26

**Authors:** Amirah Faizah Abdul Muthalib, Ibrahim Baba, Mohamed Ibrahim Mohamed Tahir, Edward R. T. Tiekink

**Affiliations:** a School of Chemical Sciences and Food Technology, Faculty of Science and Technology, Universiti Kebangsaan Malaysia, 43600 Bangi, Malaysia; b Department of Chemistry, Universiti Putra Malaysia, 43400 Serdang, Malaysia; c Department of Chemistry, University of Malaya, 50603 Kuala Lumpur, Malaysia

## Abstract

The Sn^IV^ atom in the title diorganotin dithio­carbamate, [Sn(C_4_H_9_)_2_(C_15_H_14_NS_2_)Cl], is penta­coordinated by an asymmetrically coordinating dithio­carbamate ligand, a Cl atom and two C atoms of the Sn-bound *tert*-butyl groups. The resulting C_2_ClS_2_ donor set defines a coordination geometry inter­mediate between square pyramidal and trigonal bipyramidal with a slight tendency towards the former.

## Related literature

For a review on the applications and structural chemistry of tin dithio­carbamates, see: Tiekink (2008 ▶). For additional structural analysis, see: Addison *et al.* (1984 ▶); Spek (2009 ▶). For a recently reported related structure, see: Abdul Muthalib *et al.* (2010 ▶).
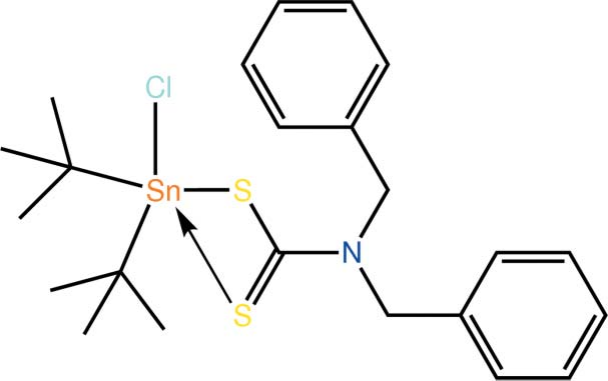


## Experimental

### 

#### Crystal data

[Sn(C_4_H_9_)_2_(C_15_H_14_NS_2_)Cl]
*M* *_r_* = 540.76
Monoclinic, 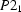

*a* = 9.0600 (2) Å
*b* = 10.9238 (2) Å
*c* = 12.7845 (3) Å
β = 102.759 (2)°
*V* = 1234.03 (5) Å^3^
*Z* = 2
Mo *K*α radiation
μ = 1.32 mm^−1^
*T* = 150 K
0.26 × 0.15 × 0.06 mm

#### Data collection

Oxford Diffraction Xcaliber Eos Gemini diffractometer
Absorption correction: multi-scan (*CrysAlis PRO*; Oxford Diffraction, 2010 ▶) *T*_min_ = 0.820, *T*_max_ = 0.924
15537 measured reflections
5443 independent reflections
5087 reflections with *I* > 2σ(*I*)
*R*_int_ = 0.046

#### Refinement

*R*[*F* ^2^ > 2σ(*F* ^2^)] = 0.032
*wR*(*F* ^2^) = 0.072
*S* = 1.05
5443 reflections
259 parameters
1 restraint
H-atom parameters constrained
Δρ_max_ = 0.78 e Å^−3^
Δρ_min_ = −0.66 e Å^−3^
Absolute structure: Flack (1983 ▶), 2497 Friedel pairs
Flack parameter: −0.035 (18)

### 

Data collection: *CrysAlis PRO* (Oxford Diffraction, 2010 ▶); cell refinement: *CrysAlis PRO*; data reduction: *CrysAlis PRO*; program(s) used to solve structure: *SHELXS97* (Sheldrick, 2008 ▶); program(s) used to refine structure: *SHELXL97* (Sheldrick, 2008 ▶); molecular graphics: *ORTEP-3* (Farrugia, 1997 ▶); software used to prepare material for publication: *publCIF* (Westrip, 2010 ▶).

## Supplementary Material

Crystal structure: contains datablocks global, I. DOI: 10.1107/S1600536811006866/pk2305sup1.cif

Structure factors: contains datablocks I. DOI: 10.1107/S1600536811006866/pk2305Isup2.hkl

Additional supplementary materials:  crystallographic information; 3D view; checkCIF report

## Figures and Tables

**Table 1:** Selected bond lengths (Å)

Sn—Cl1	2.4942 (9)
Sn—S1	2.4857 (10)
Sn—S2	2.7366 (10)
Sn—C16	2.191 (4)
Sn—C20	2.188 (3)

**Fig. 1.**
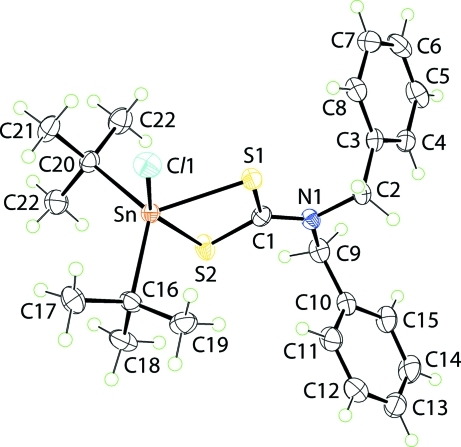
The molecular structure of of (I) showing the atom-labelling scheme and displacement ellipsoids at the 50% probability level.

## supplementary crystallographic information

### Comment

Organotin dithiocarbamates attract attention as they exhibit properties suggesting their potential as anti-cancer agents, anti-microbials and insecticides (Tiekink, 2008). In continuation of structural studies of these systems (Abdul Muthalib *et al.*, 2010), the analysis of the title compound, (I), was undertaken.

The Sn^IV^ atom in (I) is five-coordinated, being chelated by an asymmetrically coordinating dithiocarbamate ligand, a Cl and two C atoms of the Sn-bound *tert*-butyl groups, Fig. 1 and Table 1. The disparity in the C1–S1,2 bond distances reflects the asymmetric mode of coordination observed for the dithiocarbamate ligand, Table 1.

The coordination geometry is intermediate between square pyramidal and trigonal bi-pyramidal with a very slight leaning towards the former description. This assignment is based on the value calculated for τ of 0.49 for the Sn atom, which compares to the τ values of 0.0 and 1.0 for ideal square pyramidal and trigonal bi-pyramidal geometries, respectively (Spek, 2009; Addison *et al.*, 1984). The mode of coordination of the dithiocarbamate ligand, the disposition of the ligand donor set, and the intermediate coordination geometry observed for (I) matches with the literature precedents (Tiekink, 2008).

No specific intermolecular interactions are noted in the crystal packing.

### Experimental

The title compound was prepared using an *in situ* method by addition of carbon disulfide (0.01 mol) to an ethanolic solution (20 ml) of dibenzylamine (0.01 mol). The mixture was stirred for 1 h at 277 K. The resulting solution was then added drop wise to a solution of di-*tert*-butyltin(IV) dichloride (0.005 mol) in ethanol (20 ml) and stirred again for 1 h. The white precipitate was filtered, washed with cold ethanol and dried in a desiccator. Crystallization was from its ethanol:chloroform (1:2) solution. Yield 71%; *M*.pt. 475–477 K. Elemental analysis. Found (calculated) for C_23_H_32_ClNS_2_Sn: C, 50.94 (51.50); H 5.89 (5.92); N 2.59 (2.93); S 11.59 (11.86); Sn 21.25 (21.90) %. UV (CHCl_3_) λ_max_ 228 (*L*(π) →*L*(π*)). IR(KBr): ν(C—H) 2939*m*, 2849*m*; ν(C≐N) 1487*m*; ν(N—C) 1154 s; ν(C≐S) 988 s; ν(Sn—S) 351 s cm^-1^.

### Refinement

Carbon-bound H-atoms were placed in calculated positions (C—H 0.95 to 0.99 Å) and were included in the refinement in the riding model approximation, with *U*_iso_(H) set to 1.2 to 1.5*U*_equiv_(C).

### Crystal data

**Table:**

[Sn(C_4_H_9_)_2_(C_15_H_14_NS_2_)Cl]	*F*(000) = 552
*M**_r_* = 540.76	*D*_x_ = 1.455 Mg m^−^^3^
Monoclinic, *P*2_1_	Mo *K*α radiation, λ = 0.71073 Å
Hall symbol: P 2yb	Cell parameters from 10382 reflections
*a* = 9.0600 (2) Å	θ = 2.0–29.0°
*b* = 10.9238 (2) Å	µ = 1.32 mm^−^^1^
*c* = 12.7845 (3) Å	*T* = 150 K
β = 102.759 (2)°	Prism, colourless
*V* = 1234.03 (5) Å^3^	0.26 × 0.15 × 0.06 mm
*Z* = 2	

### Data collection

**Table:**

Oxford Diffraction Xcaliber Eos Gemini diffractometer	5443 independent reflections
Radiation source: fine-focus sealed tube	5087 reflections with *I* > 2σ(*I*)
graphite	*R*_int_ = 0.046
Detector resolution: 16.1952 pixels mm^-1^	θ_max_ = 27.5°, θ_min_ = 2.3°
ω scans	*h* = −11→11
Absorption correction: multi-scan (*CrysAlis PRO*; Oxford Diffraction, 2010)	*k* = −14→13
*T*_min_ = 0.820, *T*_max_ = 0.924	*l* = −16→16
15537 measured reflections	

### Refinement

**Table:**

Refinement on *F*^2^	Secondary atom site location: difference Fourier map
Least-squares matrix: full	Hydrogen site location: inferred from neighbouring sites
*R*[*F*^2^ > 2σ(*F*^2^)] = 0.032	H-atom parameters constrained
*wR*(*F*^2^) = 0.072	*w* = 1/[σ^2^(*F*_o_^2^) + (0.0329*P*)^2^] where *P* = (*F*_o_^2^ + 2*F*_c_^2^)/3
*S* = 1.05	(Δ/σ)_max_ = 0.001
5443 reflections	Δρ_max_ = 0.78 e Å^−^^3^
259 parameters	Δρ_min_ = −0.66 e Å^−^^3^
1 restraint	Absolute structure: Flack (1983), 2497 Friedel pairs
Primary atom site location: structure-invariant direct methods	Flack parameter: −0.035 (18)

### Special details

**Table:**

Geometry. All s.u.'s (except the s.u. in the dihedral angle between two l.s. planes) are estimated using the full covariance matrix. The cell s.u.'s are taken into account individually in the estimation of s.u.'s in distances, angles and torsion angles; correlations between s.u.'s in cell parameters are only used when they are defined by crystal symmetry. An approximate (isotropic) treatment of cell s.u.'s is used for estimating s.u.'s involving l.s. planes.
Refinement. Refinement of *F*^2^ against ALL reflections. The weighted *R*-factor *wR* and goodness of fit *S* are based on *F*^2^, conventional *R*-factors *R* are based on *F*, with *F* set to zero for negative *F*^2^. The threshold expression of *F*^2^ > 2σ(*F*^2^) is used only for calculating *R*-factors(gt) *etc*. and is not relevant to the choice of reflections for refinement. *R*-factors based on *F*^2^ are statistically about twice as large as those based on *F*, and *R*- factors based on ALL data will be even larger.

### Fractional atomic coordinates and isotropic or equivalent isotropic displacement parameters (Å^2^)

**Table:**

	*x*	*y*	*z*	*U*_iso_*/*U*_eq_	
Sn	0.25541 (2)	0.701510 (17)	0.187046 (15)	0.02052 (7)	
Cl1	0.25531 (12)	0.53028 (10)	0.31609 (8)	0.0317 (2)	
S1	0.30831 (12)	0.83033 (9)	0.35137 (8)	0.0254 (2)	
S2	0.30370 (12)	0.94068 (9)	0.14035 (8)	0.0306 (2)	
N1	0.3893 (3)	1.0614 (3)	0.3258 (2)	0.0234 (6)	
C1	0.3381 (4)	0.9583 (3)	0.2768 (3)	0.0238 (8)	
C2	0.4241 (4)	1.0768 (3)	0.4447 (3)	0.0238 (8)	
H2A	0.5187	1.1245	0.4670	0.029*	
H2B	0.4411	0.9952	0.4791	0.029*	
C3	0.2986 (5)	1.1410 (4)	0.4836 (4)	0.0242 (10)	
C4	0.2986 (6)	1.2679 (4)	0.4919 (4)	0.0280 (11)	
H4	0.3768	1.3142	0.4720	0.034*	
C5	0.1848 (6)	1.3273 (5)	0.5292 (4)	0.0354 (12)	
H5	0.1847	1.4141	0.5342	0.043*	
C6	0.0719 (7)	1.2598 (5)	0.5588 (4)	0.0389 (13)	
H6	−0.0061	1.3007	0.5841	0.047*	
C7	0.0709 (6)	1.1341 (6)	0.5523 (4)	0.0409 (13)	
H7	−0.0061	1.0881	0.5740	0.049*	
C8	0.1841 (6)	1.0751 (5)	0.5135 (4)	0.0325 (11)	
H8	0.1827	0.9884	0.5074	0.039*	
C9	0.4247 (4)	1.1692 (3)	0.2665 (3)	0.0295 (9)	
H9A	0.3846	1.2436	0.2948	0.035*	
H9B	0.3737	1.1610	0.1899	0.035*	
C10	0.5930 (4)	1.1841 (4)	0.2756 (3)	0.0268 (9)	
C11	0.6844 (5)	1.0857 (4)	0.2644 (4)	0.0343 (11)	
H11	0.6415	1.0061	0.2527	0.041*	
C12	0.8369 (6)	1.1017 (5)	0.2698 (4)	0.0401 (12)	
H12	0.8984	1.0328	0.2634	0.048*	
C13	0.9007 (5)	1.2160 (6)	0.2844 (3)	0.0426 (12)	
H13	1.0054	1.2268	0.2864	0.051*	
C14	0.8115 (6)	1.3152 (5)	0.2963 (4)	0.0426 (13)	
H14	0.8549	1.3947	0.3062	0.051*	
C15	0.6585 (6)	1.2997 (4)	0.2939 (4)	0.0347 (11)	
H15	0.5986	1.3680	0.3047	0.042*	
C16	0.4451 (4)	0.6314 (3)	0.1240 (3)	0.0264 (8)	
C17	0.4057 (5)	0.5006 (4)	0.0873 (4)	0.0399 (11)	
H17A	0.3221	0.5014	0.0239	0.060*	
H17B	0.3754	0.4548	0.1451	0.060*	
H17C	0.4943	0.4615	0.0694	0.060*	
C18	0.4710 (4)	0.7083 (6)	0.0303 (3)	0.0384 (9)	
H18A	0.5510	0.6709	0.0003	0.058*	
H18B	0.5016	0.7912	0.0553	0.058*	
H18C	0.3772	0.7122	−0.0252	0.058*	
C19	0.5852 (4)	0.6347 (4)	0.2167 (3)	0.0345 (10)	
H19A	0.6711	0.5977	0.1934	0.052*	
H19B	0.5646	0.5887	0.2778	0.052*	
H19C	0.6092	0.7198	0.2381	0.052*	
C20	0.0212 (3)	0.6974 (5)	0.0950 (2)	0.0256 (6)	
C21	−0.0476 (5)	0.5706 (4)	0.1105 (4)	0.0349 (11)	
H21A	−0.0504	0.5597	0.1861	0.052*	
H21B	0.0147	0.5061	0.0889	0.052*	
H21C	−0.1506	0.5657	0.0663	0.052*	
C22	0.0276 (4)	0.7156 (5)	−0.0218 (3)	0.0379 (10)	
H22A	−0.0755	0.7231	−0.0655	0.057*	
H22B	0.0776	0.6451	−0.0464	0.057*	
H22C	0.0846	0.7902	−0.0288	0.057*	
C23	−0.0699 (5)	0.7973 (4)	0.1328 (4)	0.0368 (11)	
H23A	−0.0221	0.8767	0.1269	0.055*	
H23B	−0.0737	0.7824	0.2078	0.055*	
H23C	−0.1729	0.7975	0.0883	0.055*	

### Atomic displacement parameters (Å^2^)

**Table:**

	*U*^11^	*U*^22^	*U*^33^	*U*^12^	*U*^13^	*U*^23^
Sn	0.02131 (11)	0.01858 (11)	0.02188 (12)	−0.00037 (13)	0.00521 (8)	−0.00032 (13)
Cl1	0.0404 (6)	0.0249 (5)	0.0314 (6)	0.0007 (4)	0.0112 (5)	0.0098 (4)
S1	0.0309 (5)	0.0229 (5)	0.0229 (5)	−0.0029 (4)	0.0071 (4)	−0.0003 (4)
S2	0.0448 (6)	0.0238 (5)	0.0221 (5)	−0.0044 (4)	0.0052 (4)	0.0002 (4)
N1	0.0296 (16)	0.0181 (14)	0.0230 (16)	−0.0007 (13)	0.0071 (13)	−0.0008 (12)
C1	0.0210 (17)	0.0223 (19)	0.027 (2)	0.0001 (15)	0.0031 (15)	0.0009 (15)
C2	0.0272 (19)	0.0202 (18)	0.0226 (19)	0.0006 (15)	0.0026 (16)	−0.0022 (15)
C3	0.029 (2)	0.020 (2)	0.022 (2)	−0.0015 (19)	0.0013 (19)	−0.0019 (18)
C4	0.031 (3)	0.027 (2)	0.025 (2)	−0.004 (2)	0.004 (2)	0.000 (2)
C5	0.047 (3)	0.027 (2)	0.031 (3)	0.018 (2)	0.005 (2)	−0.0026 (19)
C6	0.042 (3)	0.051 (3)	0.026 (3)	0.015 (3)	0.011 (2)	−0.004 (2)
C7	0.032 (3)	0.059 (4)	0.033 (3)	−0.005 (3)	0.010 (2)	−0.005 (3)
C8	0.039 (3)	0.032 (2)	0.026 (2)	0.000 (2)	0.007 (2)	−0.003 (2)
C9	0.040 (2)	0.020 (2)	0.027 (2)	0.0029 (14)	0.0044 (17)	0.0019 (13)
C10	0.0390 (19)	0.021 (2)	0.0202 (17)	−0.0046 (18)	0.0064 (14)	0.0024 (16)
C11	0.041 (3)	0.023 (2)	0.040 (3)	−0.0027 (19)	0.013 (2)	−0.005 (2)
C12	0.043 (3)	0.041 (3)	0.038 (3)	0.001 (2)	0.014 (2)	−0.008 (2)
C13	0.042 (2)	0.059 (3)	0.028 (2)	−0.018 (3)	0.0092 (17)	−0.001 (3)
C14	0.052 (3)	0.031 (2)	0.044 (3)	−0.020 (2)	0.010 (2)	−0.002 (2)
C15	0.046 (3)	0.023 (2)	0.033 (3)	−0.005 (2)	0.005 (2)	0.0005 (18)
C16	0.0282 (19)	0.0257 (19)	0.028 (2)	0.0031 (16)	0.0110 (17)	−0.0038 (16)
C17	0.044 (3)	0.028 (2)	0.049 (3)	0.0033 (19)	0.015 (2)	−0.0148 (19)
C18	0.0387 (19)	0.049 (2)	0.032 (2)	0.008 (3)	0.0173 (16)	−0.001 (3)
C19	0.025 (2)	0.038 (2)	0.042 (3)	0.0033 (18)	0.0096 (18)	0.000 (2)
C20	0.0210 (14)	0.0275 (16)	0.0262 (16)	0.000 (2)	0.0010 (12)	−0.001 (2)
C21	0.029 (2)	0.030 (2)	0.043 (3)	−0.0082 (19)	0.0025 (19)	−0.004 (2)
C22	0.0338 (19)	0.048 (3)	0.031 (2)	−0.001 (2)	0.0042 (15)	−0.004 (2)
C23	0.028 (2)	0.034 (3)	0.045 (3)	0.0035 (19)	0.000 (2)	−0.001 (2)

### Geometric parameters (Å, °)

**Table:**

Sn—Cl1	2.4942 (9)	C12—C13	1.372 (7)
Sn—S1	2.4857 (10)	C12—H12	0.9500
Sn—S2	2.7366 (10)	C13—C14	1.380 (8)
Sn—C16	2.191 (4)	C13—H13	0.9500
Sn—C20	2.188 (3)	C14—C15	1.389 (8)
S1—C1	1.746 (4)	C14—H14	0.9500
S2—C1	1.714 (4)	C15—H15	0.9500
N1—C1	1.321 (5)	C16—C17	1.521 (5)
N1—C9	1.474 (5)	C16—C18	1.524 (6)
N1—C2	1.493 (4)	C16—C19	1.533 (6)
C2—C3	1.510 (6)	C17—H17A	0.9800
C2—H2A	0.9900	C17—H17B	0.9800
C2—H2B	0.9900	C17—H17C	0.9800
C3—C8	1.384 (6)	C18—H18A	0.9800
C3—C4	1.390 (4)	C18—H18B	0.9800
C4—C5	1.389 (6)	C18—H18C	0.9800
C4—H4	0.9500	C19—H19A	0.9800
C5—C6	1.381 (8)	C19—H19B	0.9800
C5—H5	0.9500	C19—H19C	0.9800
C6—C7	1.376 (5)	C20—C23	1.510 (6)
C6—H6	0.9500	C20—C22	1.519 (5)
C7—C8	1.392 (7)	C20—C21	1.550 (6)
C7—H7	0.9500	C21—H21A	0.9800
C8—H8	0.9500	C21—H21B	0.9800
C9—C10	1.512 (5)	C21—H21C	0.9800
C9—H9A	0.9900	C22—H22A	0.9800
C9—H9B	0.9900	C22—H22B	0.9800
C10—C11	1.383 (6)	C22—H22C	0.9800
C10—C15	1.394 (6)	C23—H23A	0.9800
C11—C12	1.380 (7)	C23—H23B	0.9800
C11—H11	0.9500	C23—H23C	0.9800
			
C20—Sn—C16	122.78 (14)	C12—C13—C14	119.3 (4)
C20—Sn—S1	116.70 (11)	C12—C13—H13	120.3
C16—Sn—S1	119.21 (10)	C14—C13—H13	120.3
C20—Sn—Cl1	101.46 (13)	C13—C14—C15	120.5 (4)
C16—Sn—Cl1	95.51 (10)	C13—C14—H14	119.7
S1—Sn—Cl1	83.90 (4)	C15—C14—H14	119.7
C20—Sn—S2	94.87 (14)	C14—C15—C10	120.0 (4)
C16—Sn—S2	94.43 (10)	C14—C15—H15	120.0
S1—Sn—S2	68.51 (3)	C10—C15—H15	120.0
Cl1—Sn—S2	152.09 (3)	C17—C16—C18	110.0 (4)
C1—S1—Sn	90.91 (13)	C17—C16—C19	111.0 (3)
C1—S2—Sn	83.51 (12)	C18—C16—C19	110.5 (3)
C1—N1—C9	122.1 (3)	C17—C16—Sn	107.0 (3)
C1—N1—C2	123.5 (3)	C18—C16—Sn	111.5 (3)
C9—N1—C2	114.4 (3)	C19—C16—Sn	106.9 (2)
N1—C1—S2	122.9 (3)	C16—C17—H17A	109.5
N1—C1—S1	120.3 (3)	C16—C17—H17B	109.5
S2—C1—S1	116.8 (2)	H17A—C17—H17B	109.5
N1—C2—C3	112.5 (3)	C16—C17—H17C	109.5
N1—C2—H2A	109.1	H17A—C17—H17C	109.5
C3—C2—H2A	109.1	H17B—C17—H17C	109.5
N1—C2—H2B	109.1	C16—C18—H18A	109.5
C3—C2—H2B	109.1	C16—C18—H18B	109.5
H2A—C2—H2B	107.8	H18A—C18—H18B	109.5
C8—C3—C4	119.0 (5)	C16—C18—H18C	109.5
C8—C3—C2	120.9 (4)	H18A—C18—H18C	109.5
C4—C3—C2	120.1 (5)	H18B—C18—H18C	109.5
C5—C4—C3	120.3 (5)	C16—C19—H19A	109.5
C5—C4—H4	119.8	C16—C19—H19B	109.5
C3—C4—H4	119.8	H19A—C19—H19B	109.5
C6—C5—C4	119.7 (5)	C16—C19—H19C	109.5
C6—C5—H5	120.1	H19A—C19—H19C	109.5
C4—C5—H5	120.1	H19B—C19—H19C	109.5
C7—C6—C5	120.7 (6)	C23—C20—C22	111.2 (4)
C7—C6—H6	119.6	C23—C20—C21	109.9 (3)
C5—C6—H6	119.6	C22—C20—C21	110.3 (4)
C6—C7—C8	119.3 (6)	C23—C20—Sn	110.3 (3)
C6—C7—H7	120.4	C22—C20—Sn	106.5 (2)
C8—C7—H7	120.4	C21—C20—Sn	108.6 (3)
C3—C8—C7	120.9 (5)	C20—C21—H21A	109.5
C3—C8—H8	119.6	C20—C21—H21B	109.5
C7—C8—H8	119.6	H21A—C21—H21B	109.5
N1—C9—C10	112.1 (3)	C20—C21—H21C	109.5
N1—C9—H9A	109.2	H21A—C21—H21C	109.5
C10—C9—H9A	109.2	H21B—C21—H21C	109.5
N1—C9—H9B	109.2	C20—C22—H22A	109.5
C10—C9—H9B	109.2	C20—C22—H22B	109.5
H9A—C9—H9B	107.9	H22A—C22—H22B	109.5
C11—C10—C15	118.6 (4)	C20—C22—H22C	109.5
C11—C10—C9	121.7 (4)	H22A—C22—H22C	109.5
C15—C10—C9	119.7 (4)	H22B—C22—H22C	109.5
C12—C11—C10	120.8 (4)	C20—C23—H23A	109.5
C12—C11—H11	119.6	C20—C23—H23B	109.5
C10—C11—H11	119.6	H23A—C23—H23B	109.5
C13—C12—C11	120.6 (5)	C20—C23—H23C	109.5
C13—C12—H12	119.7	H23A—C23—H23C	109.5
C11—C12—H12	119.7	H23B—C23—H23C	109.5
			
C20—Sn—S1—C1	−87.69 (18)	C15—C10—C11—C12	−0.7 (6)
C16—Sn—S1—C1	79.62 (17)	C9—C10—C11—C12	178.1 (4)
Cl1—Sn—S1—C1	172.53 (12)	C10—C11—C12—C13	−1.3 (7)
S2—Sn—S1—C1	−3.20 (12)	C11—C12—C13—C14	1.5 (7)
C20—Sn—S2—C1	120.10 (15)	C12—C13—C14—C15	0.2 (7)
C16—Sn—S2—C1	−116.43 (16)	C13—C14—C15—C10	−2.3 (7)
S1—Sn—S2—C1	3.28 (12)	C11—C10—C15—C14	2.5 (6)
Cl1—Sn—S2—C1	−5.82 (15)	C9—C10—C15—C14	−176.3 (4)
C9—N1—C1—S2	−1.6 (5)	C20—Sn—C16—C17	−53.6 (3)
C2—N1—C1—S2	−178.1 (3)	S1—Sn—C16—C17	139.9 (2)
C9—N1—C1—S1	176.3 (3)	Cl1—Sn—C16—C17	53.8 (3)
C2—N1—C1—S1	−0.2 (5)	S2—Sn—C16—C17	−152.3 (3)
Sn—S2—C1—N1	173.1 (3)	C20—Sn—C16—C18	66.6 (3)
Sn—S2—C1—S1	−4.87 (18)	S1—Sn—C16—C18	−99.9 (3)
Sn—S1—C1—N1	−172.7 (3)	Cl1—Sn—C16—C18	174.0 (3)
Sn—S1—C1—S2	5.3 (2)	S2—Sn—C16—C18	−32.1 (3)
C1—N1—C2—C3	−99.7 (4)	C20—Sn—C16—C19	−172.6 (3)
C9—N1—C2—C3	83.5 (4)	S1—Sn—C16—C19	20.9 (3)
N1—C2—C3—C8	91.7 (5)	Cl1—Sn—C16—C19	−65.1 (3)
N1—C2—C3—C4	−89.6 (5)	S2—Sn—C16—C19	88.7 (3)
C8—C3—C4—C5	−0.3 (9)	C16—Sn—C20—C23	−154.4 (3)
C2—C3—C4—C5	−179.0 (3)	S1—Sn—C20—C23	12.4 (3)
C3—C4—C5—C6	0.5 (8)	Cl1—Sn—C20—C23	101.3 (3)
C4—C5—C6—C7	0.2 (9)	S2—Sn—C20—C23	−55.9 (3)
C5—C6—C7—C8	−1.1 (10)	C16—Sn—C20—C22	−33.6 (5)
C4—C3—C8—C7	−0.6 (8)	S1—Sn—C20—C22	133.2 (3)
C2—C3—C8—C7	178.1 (4)	Cl1—Sn—C20—C22	−137.9 (4)
C6—C7—C8—C3	1.3 (9)	S2—Sn—C20—C22	64.9 (4)
C1—N1—C9—C10	−102.5 (4)	C16—Sn—C20—C21	85.1 (3)
C2—N1—C9—C10	74.3 (4)	S1—Sn—C20—C21	−108.0 (3)
N1—C9—C10—C11	44.6 (5)	Cl1—Sn—C20—C21	−19.2 (3)
N1—C9—C10—C15	−136.6 (4)	S2—Sn—C20—C21	−176.4 (2)
